# Teachers’ perceived time pressure, emotional exhaustion and the role of social support from the school principal

**DOI:** 10.1007/s11218-020-09605-8

**Published:** 2021-03-11

**Authors:** Jasper Maas, Simone Schoch, Urte Scholz, Pamela Rackow, Julia Schüler, Mirko Wegner, Roger Keller

**Affiliations:** 1 Leiden Institute of Education and Child Studies, Leiden University, Wassenaarseweg 52, 2333 AK, Leiden, Netherlands; 2 Centre for Inclusion and Health in Schools, Zurich University of Teacher Education, Lagerstrasse 2, 8090 Zürich, Switzerland; 3 Department of Psychology, Applied Social and Health Psychology, University of Zurich, Binzmühlestrasse 14 / Box 14, 8050 Zürich, Switzerland; 4 Faculty of Natural Sciences, Psychology, University of Stirling, Stirling, FK9 4LA UK; 5 Department of Sports Science, Sport Psychology, University of Konstanz, Universitätsstrasse 10, 78464 Konstanz, Germany; 6 Faculty of Humanities and Social Sciences, Sport Psychology, Humboldt-Universität Zu Berlin, Sports Sciences, Philippstrasse 13, Haus 11, 10115 Berlin, Germany

**Keywords:** Time pressure, Emotional exhaustion, Social support, School principals, Job demands-resources model

## Abstract

Many teachers experience high levels of work-related strain due to time pressure, which over time can lead to various health problems, such as emotional exhaustion. However, there is growing evidence that this could be a reciprocal effect. Moreover, it is known that perceived social support can buffer the negative effects of stress, such as time pressure, on health outcomes. Less is known about buffering effects of received social support. Based on longitudinal data of *n* = 1071 Swiss primary and secondary school teachers over the course of one school year, the present study examined the reciprocal relationship between teachers’ perceived time pressure and emotional exhaustion and whether received social support from the school principal buffers this relationship. Results of a random intercept cross-lagged panel model show a strong relationship between teachers’ perceived time pressure and emotional exhaustion at the between-person level, but no effects at the within-person level. Further, received social support was directly related to less perceived time pressure and less emotional exhaustion. The results showed neither evidence for reciprocal effects between perceived time pressure and emotional exhaustion nor for a buffering effect of received social support from the school principal. Concluding, present findings indicate that the receipt of social support from the school principal is a central job resource that beneficially relates to teachers’ experience of time pressure and emotional exhaustion.

## Introduction

To meet their professional demands, it is important that teachers remain motivated and healthy at their work. However, many teachers experience high work pressure, lack of recovery time, and exhaustion (Sandmeier et al. 2017). Impaired teachers’ health may have severe consequences such as reduced teaching quality (Klusmann et al. 2008), reduced job satisfaction (Skaalvik and Skaalvik 2017c), higher intention to leave the profession (Skaalvik and Skaalvik 2017c), and lower students’ achievement (Klusmann et al. 2016). Hence, teachers’ health is an essential precondition to fulfil the educational mandate (Nieskens 2006; Sieland 2006). Promoting and maintaining teachers’ health, therefore, is one of the many challenges schools are facing (Harazd et al. 2009) to which the present study aims to contribute by expanding the understanding of the relationship between time pressure and emotional exhaustion and the role of social support.

The Job Demands-Resources model (JD-R model; Bakker and Demerouti 2017) proposes that a reciprocal process takes place between job demands and health complaints in which they amplify each other. Social support may weaken this process by buffering detrimental effects of job demands (Bakker et al. 2005). The present study examines these assumptions for teachers’ perceived time pressure (as a central job demand for teachers, e.g., Brägger 2019), emotional exhaustion (as a health complaint; Schwarzer, Schmitz, and Tang 2000). The receipt of social support from the school principal is examined as a job resource to counter this. Following recommendations to distinguish between interindividual differences and intraindividual changes in longitudinal designs (Curran and Bauer 2011; Kievit, Frankenhuis, Waldorp, and Borsboom 2013), we apply a random intercept cross-lagged panel model (RI-CLPM; Hamaker, Kuiper, and Grasman 2015) to distinguish between interindividual differences (between-person effects) and intraindividual changes (within-person effects).

## Theoretical framework

### Time pressure and emotional exhaustion

Time pressure is characterized as the perception of a lack of available time in relation to the amount of workload, which is accompanied with the emotional experience of being rushed (Szollos 2009). This situation can result in a health impairment process as described in the JD-R model (Bakker and Demerouti 2017). Experiencing time pressure over a long time period requires long-term efforts that, in turn, are associated with physiological and/or psychological costs. Over time, these costs result in energy depletion, fatigue and health complaints (Bakker and Demerouti 2007). Indeed, previous research on teachers’ perceived time pressure emphasizes negative consequences such as burnout (Skaalvik and Skaalvik 2017b).

In the present study we focus on emotional exhaustion as the central element and most obvious manifestation of burnout (Maslach et al. 2001). Burnout refers to a psychological syndrome in response to chronic emotional and interpersonal job stressors and is defined by three dimensions: emotional exhaustion, cynicism, and inefficacy (Maslach et al. 2001). Emotional exhaustion reflects the stress component of burnout and is characterized as a lack of energy, depletion of emotional resources, chronic fatigue, and the feeling of being worn out (Schwarzer et al. 2000). Moreover, it seems that the experience of time pressure among teachers is stronger related to emotional exhaustion in comparison to the other dimensions depersonalization (cynicism) and inefficacy (Skaalvik and Skaalvik 2009, 2017a; van Droogenbroeck, Spruyt, and Vanroelen 2014).

### Time pressure and emotional exhaustion: a reciprocal process?

According to the JD-R model the relationship between job demands and impaired health is reciprocal. Not only can job demands (e.g., time pressure) predict impaired health (e.g., emotional exhaustion), but conversely, an increase in job demands can also be a consequence of impaired health (Bakker and Demerouti 2017). The underlying mechanism that explains the latter reversed process has been described as self-undermining (Bakker and Costa 2014). In reaction to high strain, employees may lose self-regulatory resources, display dysfunctional behaviours, and therefore create obstacles which may undermine their performance (Bakker and Wang 2019). Employees who experience emotional exhaustion can for example exhibit self-undermining behaviours such as poor communication, making mistakes, and creating conflicts which add up to already existing job demands (Bakker and Costa 2014; Bakker and Wang 2019). Hence, teachers that experience time pressure may feel emotionally exhausted which, in turn, leads to dysfunctional behaviour such as working inefficiently, resulting in even more time pressure. This reciprocal relationship between time pressure and emotional exhaustion may even result in a loss cycle (Hobfoll 2001).

Previous research on reciprocal models of job demands and employees’ health supports the assumption of reciprocity (e.g., de Lange et al. 2004; van der Heijden, Demerouti and Bakker 2008). Compared to causal models (job demands predict health impairments) and reversed causation models (health impairments predict job demand), reciprocal models of job demands and burnout perform better (Lesener et al. 2019). Moreover, specific reciprocal models of emotional exhaustion and job demands related to time pressure (i.e., work pressure, work overload, work hours), also reflect reciprocal relationships (Demerouti et al. 2004; ten Brummelhuis et al. 2011).

### The role of social support

**Fig. 1:**
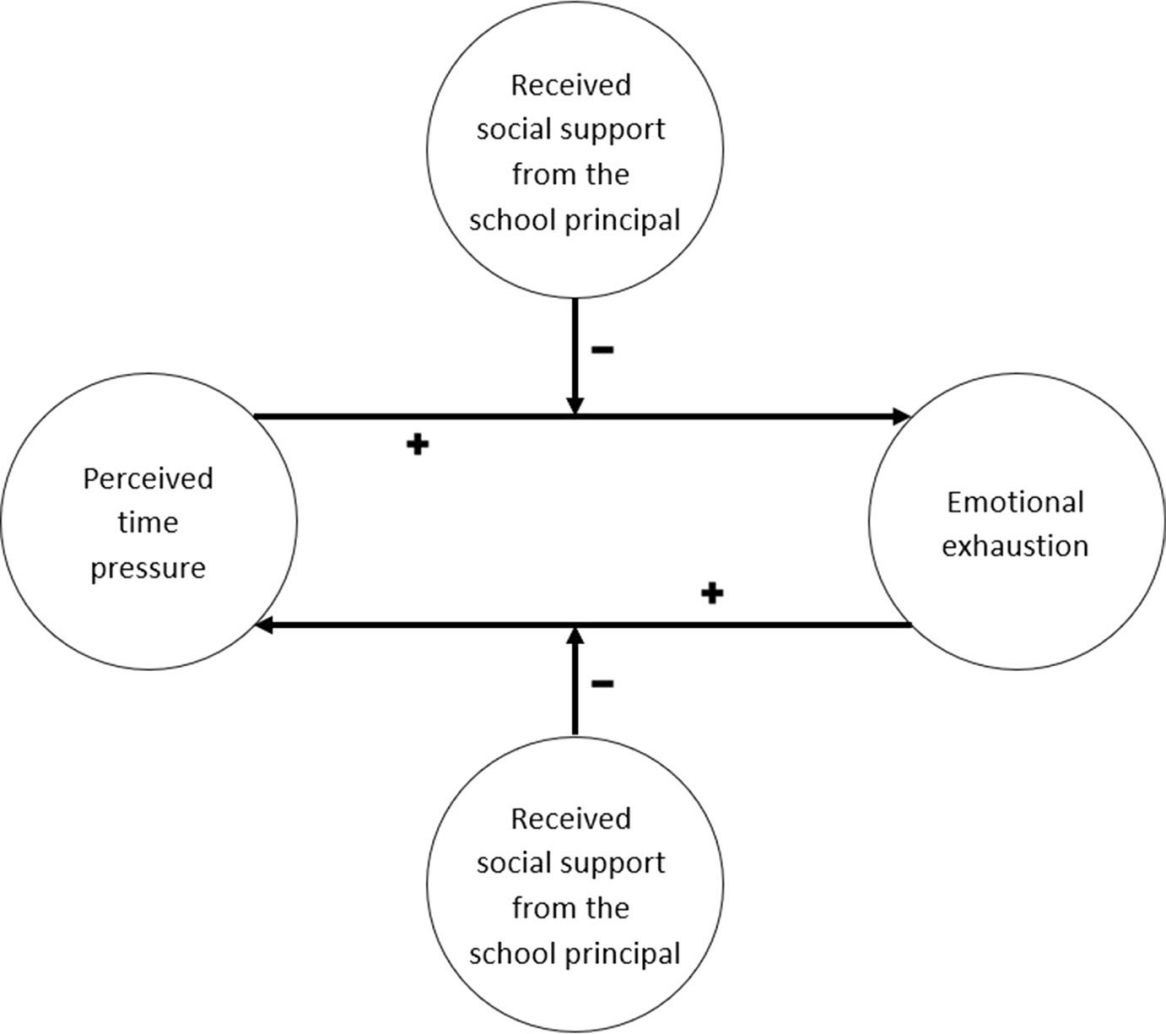
Conceptual research model

The present study examines whether social support buffers the reciprocal relationship between perceived time pressure and emotional exhaustion (see Fig. 1). Social support refers to the qualitative aspect of social interactions that can influence a problematic situation or improve coping with it (Knoll and Kienle 2007). The difference between perceived and received social support is an important distinction in social support research. *Perceived* or *anticipated social support* describes the support that a person thinks is potentially available in their social network when help is needed (Knoll and Kienle 2007). Several studies indicated that perceived social support is rather a stable than a modifiable characteristic (e.g., Sarason et al. 1987). Thus, *perceived* social support is somewhat independent from the behaviour of a specific network member and therefore not a good indicator for supportive interactions (Knoll and Kienle 2007). In contrast, *received social support* is the retrospective report of actual support transactions from specific network members (Uchino 2009). Receiving social support might even be associated with lower self-esteem and threaten one’s feeling of independence if there is no need for social support (Uchino 2009). Whereas perceived social support has almost solely favourable effects (Cohen 2004), research on received support demonstrates mixed results including negative effects on health and well-being (Beehr et al. 2010; Deelstra et al. 2003; Scholz et al. 2012).

The JD-R model considers social support as a job resource that can contribute to achieving work goals, reducing job demands and the associated physiological and psychological costs, or stimulating personal growth, learning, and development (Bakker and Demerouti 2017). One mechanism through which social support can have a positive impact is described in the buffer hypothesis (Cohen 2004), which suggests that social support is most beneficial for individuals experiencing high stress. This assumption is integrated in the JD-R model by stating that several job resources, including social support, can weaken the stressful impact of several job demands (Bakker and Demerouti 2017). Indeed, social support is an important job resource associated with improved employees’ health that can buffer the effects of job demands (Bakker et al. 2005; Viswesvaran et al. 1999). Yet, little is known about buffering effects of received social support in contrast to perceived social support.

Moreover, in school settings social support was found to buffer effects of various job demands, such as the negative impact of student misbehaviour on teachers’ work engagement (Bakker et al. 2007), the positive effects of workload on teachers’ intention to leave the profession (Pomaki et al. 2010), emotional labour on teachers’ emotional exhaustion (Kinman et al. 2011), and the effects of subjective job stress on teachers’ blood pressure and heart rate (Steptoe 2000). However, to the best of our knowledge, studies on the specific buffering effects of social support from the school principal are scarce. School principals might, more than colleagues, be in the position and have the competencies to influence job demands and therefore play an important role in promoting teachers’ health (Harazd et al. 2009). This could explain why social support from the supervisor seems to be stronger related to job demands and employee outcomes than social support from colleagues (Mathieu et al. 2019). Studies on main effects of social support from the school principal show it to be negatively related to teachers’ job demands such as time pressure (Skaalvik and Skaalvik 2009), workload (Dick and Wagner 2001), teachers’ emotional exhaustion (Skaalvik and Skaalvik 2009), stress and job dissatisfaction (Bradley 2007). To better understand the possible buffering effect of received social support from the school principal on the relationship between perceived time pressure and emotional exhaustion, we focus on the social support that teachers received from their school principal.

### The aim of the present study

The aim of the present study is to examine the relationship between teachers’ perceived time pressure and emotional exhaustion as well as to test buffering effects of received social support from the school principal. The JD-R model (Bakker and Demerouti 2017) proposes a reciprocal process between job demands and health complaints. Job demands, such as time pressure, can result in energy depletion and health complaints, such as emotional exhaustion. At the same time, impaired health may generate dysfunctional behaviour which can result in an increase of job demands. Based on this we expect to find a reciprocal process between teachers’ perceived time pressure and emotional exhaustion (see Fig. 1) and hypothesize:

#### **Hypothesis 1**

Perceived time pressure positively influences emotional exhaustion.

#### **Hypothesis 2**

Emotional exhaustion positively influences perceived time pressure.

This reciprocal process has the risk of becoming a loss cycle of accumulating time pressure and emotional exhaustion. Therefore, it is important to find out how to prevent this. Based on the buffer hypothesis of social support (Cohen 2004) we propose that the receipt of social support from the school principal can buffer the reciprocal effects between time pressure and emotional exhaustion (see Fig. 1). Accordingly, we hypothesize:

#### **Hypothesis 3**

Social support from the school principal weakens the reciprocal effects between emotional exhaustion and perceived time pressure.

## Method

### Procedure

The present research examined the hypotheses in a sample of teachers at primary (pupils aged 5 to 12 years) and lower-secondary (pupils aged 13 to 15 years) compulsory school level in the German-speaking part of Switzerland. Participants were recruited through cantonal teacher organisations and they registered individually for participation by giving written informed consent. We decided against recruitment in schools to avoid a hierarchical data structure and to recruit a wide range of teachers with different school principals. Eligible for participation were teachers that met the following criteria: Teaching primary or lower secondary school level, having a minimum workload of 10 lessons per week, and working at a school with a formal school principal. Participants were asked to fill out three online questionnaires in the school year 2017/2018. The first questionnaire was administered in September 2017 at the start of the school year (T1), the second halfway through the school year in January 2018 (T2), and the third May 2018 almost at the end of the school year (T3). Following the debriefing of the participants after completing each questionnaire, participants received a voucher worth 25 Swiss francs for each completed online questionnaire as compensation for their participation.

### Participants

In total, *N* = 1365 teachers took part in the study of which 110 participants did not meet the conditions for participation. Of the remaining sample of *N* = 1255 participants, *n* = 1042 (83.0%) completed all three questionnaires. Over the course of the three measurement points *n* = 213 participants dropped out (17.0%). Independent samples t-tests of study participants who dropped out during the study (at either T2 or T3) and study participants who continued participation, demonstrated no significant differences in the model variables perceived time pressure, emotional exhaustion, and received social support from the school principal at both T1 and T2. From the total sample of *N* = 1255 participants, data of *n* = 184 participants were excluded: *n* = 154 participants working as special education teacher worked with very small groups of students in contrast to the other study participants working with whole classes. Additionally, because we are conducting longitudinal analyses, we excluded *n* = 28 participants who changed school or school principal during the school year. Moreover, we had to exclude *n* = 2 participants who gave implausible answers. The final sample consisted of *N* = 1071 teachers, 79.5% female and 18.3% male teachers (2.1% persons did not report gender). Age ranged between 22 and 65 years, with a mean of 42.8 years (SD = 11.27). Teaching level was distributed as follows: 74.7% primary school level, 23.1% lower secondary school level, and 2.2% taught both primary and secondary school level. The mean teaching experience was 17.3 years (SD = 10.86), and the mean workload 80.51% of a full-time equivalent (SD = 19.07). Although the study did not aim to obtain representative data, the sample corresponded largely to the population of teachers in the German-speaking part of Switzerland in the schoolyear 2016/17 (Federal Statistical Office 2018).

### Measures

All measurement instruments were used as part of a larger research project on school leadership and teachers’ health and assessed at every measurement point (T1–T3). Means, standard deviations, and reliability (Cronbach’s Alpha) of all measures at all three measurement points are displayed in Table 1.

**Table 1:** Means, standard deviations, internal consistencies, and correlations between all model variables (*N* = 1071)

Variable	*M*	*SD*	1	2	3	4	5	6	7	8	9
1. Time pressure T1	3.06	.94	(.74)								
2. Time pressure T2	3.04	.93	.63	(.74)							
3. Time pressure T3	3.08	.94	.63	.61	(.76)						
4. Emotional exhaustion T1	1.88	.55	.53	.40	.41	(.84)					
5. Emotional exhaustion T2	1.83	.53	.39	.52	.40	.64	(.84)				
6. Emotional exhaustion T3	1.90	.58	.44	.43	.55	.64	.64	(.86)			
7. Social support from the school principal T1	4.11	1.26	− .15	− .09	− .15	− .19	− .18	− .21	(.94)		
8. Social support from the school principal T2	4.08	1.27	− .13	− .11	− .14	− .15	− .19	− .17	.76	(.94)	
9. Social support from the school principal T3	3.93	1.31	− .19	− .15	− .20	− .17	− .21	− .22	.69	.72	(.95)

*M*, mean, *SD*, standard deviation. T1, measurement point 1, T2, measurement point 2, T3, measurement point 3. Cronbach’s alphas are listed on the diagonal in parentheses. All correlations are significant at the *p* < .01. level, except the correlation between social support T1 and time pressure T2 (*p* > .05)

*Perceived time pressure*. Perceived time pressure was assessed using the subscale “Time pressure” of the questionnaire on psychological strain among teachers in Germany (Nübling et al. 2008). The scale consists of three items. A sample item is “I was frequently under time pressure”. Response scales range from 1 (does not apply at all) to 5 (applies completely). To investigate the structure of this measure, an exploratory factor analysis was conducted which revealed one factor that explained 66.18% of the variance at T1, 65.87% at T2, and 67.89% at T3. Over all three measurement points factor loadings ranged between 0.764 and 0.861.

*Emotional exhaustion*. Emotional exhaustion was assessed using the subscale of the German version of the Maslach Burnout Inventory (Maslach, Jackson, and Leiter 1997; Schwarzer and Jerusalem 1999). The scale consists of nine items. A sample item is “Because of my work I felt exhausted”. Response scales range from 1 (is not true) to 4 (absolutely true). An exploratory factor analysis revealed one factor that explained 44.96% of the variance at T1, 44.31% at T2, and 48.44% at T3. Over all three measurement points factor loadings ranged between 0.505 and 0.804.

*Received social support from the school principal.* An adapted version of the Actually Received Support scale from the Berlin Social Support Scales was used to assess the receipt of social support from the school principal. The original scale was reformulated to fit the school setting. The instrument consists of the subscales emotional and instrumental support which are merged to create one scale, due to a strong correlation (T1: *r* = 0.85, T2: *r* = 0.84, T3: *r* = 0.86). Emotional and instrumental support are frequently stronger related within professions that are characterized by high emotional demands (Mathieu et al. 2019). Three negatively worded items were omitted from the original scale because they did not load on the intended factor but constituted a separate factor. A phenomenon that appears to be rather common for negatively worded items (Barnette 2000). This resulted in a scale consisting of ten items. A sample item is “My principal took care of things I could not manage on my own” and response scales range from 1 (is not true) to 6 (absolutely true). An exploratory factor analysis revealed one factor that explained 63.87% of the variance at T1, 64.39% at T2, and 67.31% at T3. Over all three measurement points factor loadings ranged between 0.725 and 0.879.

### Data-analytical strategy

For the main analyses we used R version 3.4.3 (R Core Team 2018) and the packages Lavaan version 0.6–3 (Rosseel 2012), Missmech version 1.0.2 (Jamshidian et al. 2014), and Multilevel version 2.6 (Bliese 2016). Multivariate normality was not affirmed in our data, therefore we estimated the model with robust maximum likelihood (MLR; Lai 2018). According to the non-parametric test of missing values completely at random (MCAR; Jamshidian et al. 2014) no sufficient evidence was found to reject MCAR. Thus, we treated missing values with full information maximum likelihood (FIML; Graham and Coffman 2012).

To assess model fit, four fit indices are reported: Chi-square/*df* ratio (*χ*^2^/df), the Root Mean Square of Approximation (RMSEA), Standardized Root Mean Residual (SRMR), and the Comparative Fit Index (CFI). Values of *χ*^2^/df less than 5 indicate good model fit (West et al. 2012). RMSEA values of ≤ 0.06 indicate a good fit, SRMR values of ≤ 0.08 are considered as a good fit, and the incremental fit indices CFI reflects good fit when above 0.95 (Hu and Bentler 1999).

To examine whether perceived time pressure and emotional exhaustion are reciprocally related, we applied the Random Intercept Cross-Lagged Panel Model (RI-CLPM; Hamaker et al. 2015). This model allows testing reciprocity while distinguishing between interindividual differences and intraindividual changes. This distinction is important in longitudinal designs to draw conclusions at the appropriate analytical level (Curran and Bauer 2011) and because within- and between-person effects do not necessarily correspond, but may even be contrary to each other (Kievit et al. 2013). Between-person effects reflect covariances in rank order positions of individuals (i.e., the level of perceived time pressure or emotional exhaustion of one specific teacher relative to all other teachers in the sample). This captures interindividual differences and not intraindividual changes (Hamaker et al. 2015). We followed the procedure of Hamaker et al. (2015) to specify the RI-CLPM and separated the variance of all model variables in stable time-invariant components at the between-person level and time-variant components at the within-person level by including random intercepts. We constrained factor loadings of the random intercepts and the within-person latent variables to 1. Further, to reduce model complexity, we used the observed indicators to calculate average scale scores that function as the observed variables in the specified models.

**Fig. 2:**
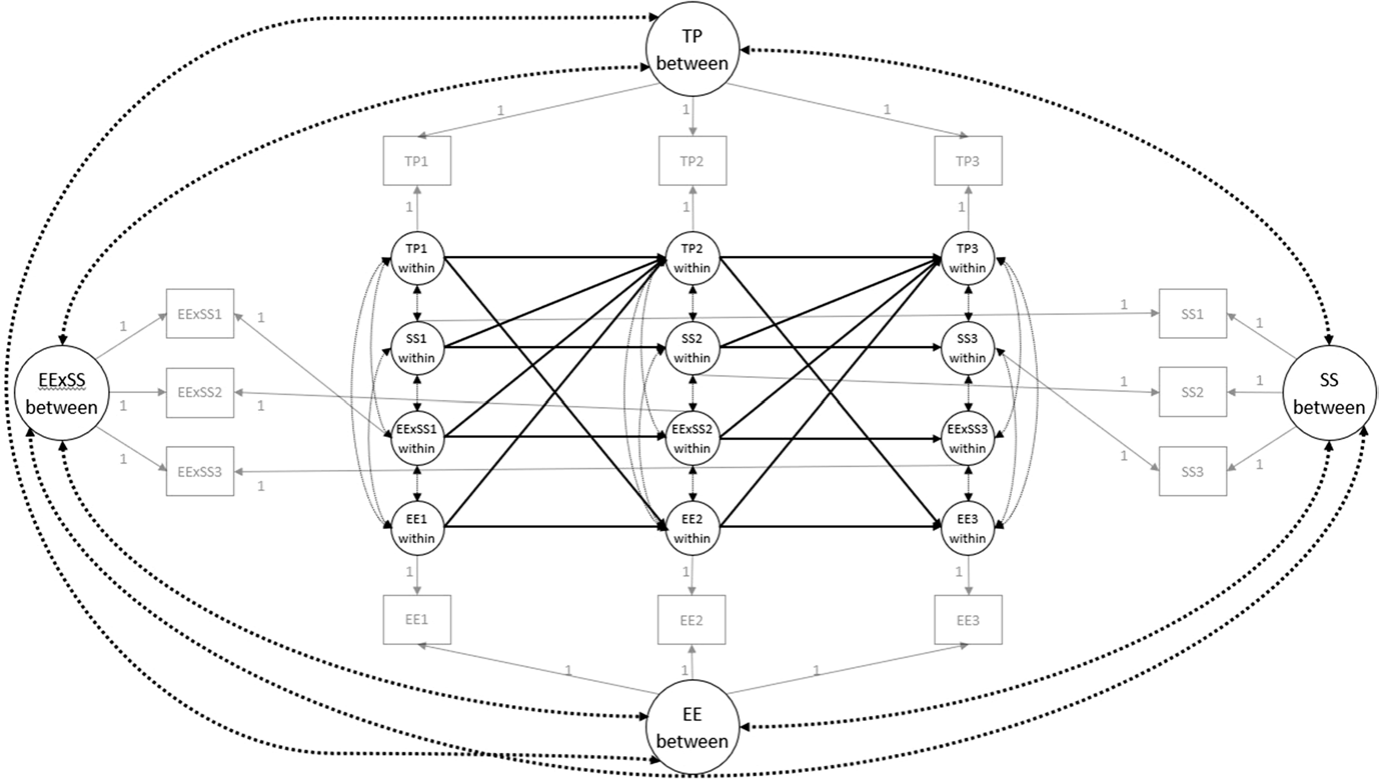
Random Intercept Cross-Lagged Panel Model, linking perceived time pressure (TP), emotional exhaustion (EE), social support (SS), and the interaction between emotional exhaustion and social support (EExSS) across three measurement points, differentiating within- and between-person variance indicated as “within” and “between”. Grey squares and lines represent scale scores and factor loadings. Dotted lines represent correlations between random intercepts and cross-sectional correlations at the within-person level. Black circles and lines represent latent variables and lagged paths. Numbers in squares and circles represent the measurement point

Buffering effects of received social support were assessed by constructing interaction terms according to the matched-pair strategy (Marsh et al. 2004) and including them in the RI-CLPM. Observed indicators were matched according to the factor loadings: The indicator with the highest factor loading from one predictor was matched with the indicator with the highest factor loading from the other predictor, and so on. If the number of indicators differed between predictors, indicators from the predictor with the highest number of items were omitted to match the number of indicators of the other predictor (e.g., for the interaction between time pressure (3 items) and social support (10 items) the three indicators of social support with the highest factor loading were used and the rest omitted). Compared to building parcels of the larger predictor, this strategy proved to perform better if the data distribution is non-normal (Wu et al. 2013). Furthermore, we applied double-mean centring by grand-mean centring each observed indicator before calculating the product indicators as well as grand-mean centring the product indicators. This strategy is recommended when normality assumptions are violated (Lin et al. 2010). The model variables time pressure (TP), emotional exhaustion (EE), and social support (SS) constituted the interaction terms TPxSS and EExSS. To test these interactions separately we specified two models that differ from each other in the interaction term: TPxSS or EExSS. Figure 2 shows one of these models in an exemplary way.

## Results

### Descriptive results

Table 1 shows means, standard deviations, bivariate correlations, and Cronbach’s alphas of the model variables across all three measurement points. Correlations between measurements of the same construct over time (T1–T3) ranged between 0.61 and 0.76. Internal consistencies for all variables at all three measurement points were satisfactory (*α* ≥ 0.74).

To determine whether applying a RI-CLPM would be appropriate, we first tested conventional cross-lagged panel models (CLPM) and compared them to the RI-CLPM with the same interaction term. The CLPM can be considered as the traditional model for testing reciprocal relationships, only with the limitation that it does not separate variance at a within- and between-person level and therefore lagged effects are not controlled for stable, time-invariant differences between persons. All RI-CLPMs fitted the data much better compared to the conventional CLPM (see Table 2). This indicated that the model variables are quite stable across the three measurement points. In a next step, we determined how much variance is due to differences between teachers and how much is due to changes within teachers across the three measurement points. Intraclass correlations (ICC) showed that 62.3% to 71.8% of the variance of perceived time pressure, emotional exhaustion, and social support was due to differences between teachers. The ICC for perceived time pressure was 0.623, for emotional exhaustion 0.634, and for social support 0.718. The variance of the two interaction terms was mainly due to changes within teachers in one school year (55.9% and 63.1%). The ICC for the interaction between emotional exhaustion and social support was 0.441 and between time pressure and social support 0.369. Concluding, all model variables reflected sufficient amounts of variance at both the within- and between-person level, which further justified the specification of a RI-CLPM.

**Table 2:** Model fit comparison of the cross-lagged panel models and random intercept cross-lagged panel models with free estimated parameters

Interaction and model	χ^2^ (df)	χ^2^/df	RMSEA	SRMR	CFI	Δ *χ*^2^ (Δdf)
*Model with interaction EExSS*						
CLPM	373.89 (32)	11.68	.100	.057	.906	
RI-CLPM	28.07 (22)	1.28	.016	.018	.998	287.10(10) ***
*Model with interaction TPxSS*						
CLPM	391.95(32)	12.25	.103	.053	.899	
RI-CLPM	30.02 (22)	1.37	.018	.019	.998	299.31(10) ***

CLPM, cross-lagged panel model. RI-CLPM, random intercept cross-lagged panel model. EExSS, interaction between emotional exhaustion and social support. TPxSS, interaction between time pressure and social support

^***^p < .001

**Fig. 3:**
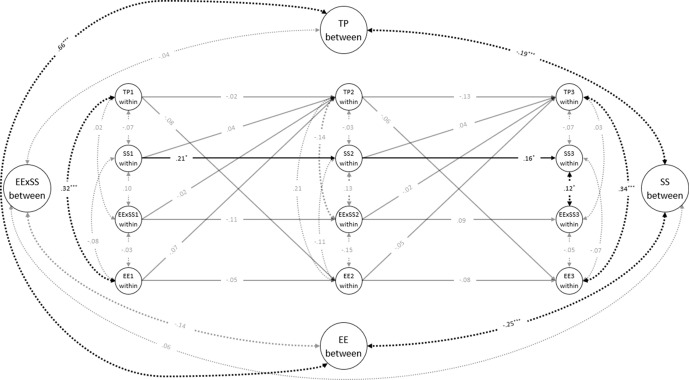
Standardized effects (*β*) of the random intercept cross-lagged panel model linking perceived time pressure (TP), emotional exhaustion (EE), received emotional support (ES), and the interaction between emotional exhaustion and emotional support (EExES), with equal cross-lagged effects over three measurement points. Numbers in squares and circles represent the measurement point. Dotted lines represent correlations and solid lines represent lagged paths. Grey lines represent insignificant paths *p* > .05, black lines represent significant paths *p* < .05. * *p* < .05, ** *p* < .010, *** *p* < .001. Model fit: χ² (df) = 34.91 (26), χ²/df = 1.34, RMSEA = .019, SRMR = .019, and CFI = .998

**Fig. 4:**
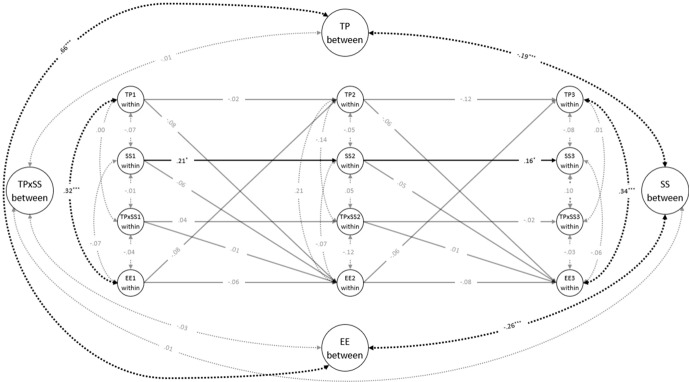
Standardized effects $(\beta)$ of the random intercept cross-lagged panel model linking perceived time pressure (TP), emotional exhaustion (EE), received instrumental support (IS), and the interaction between time pressure and instrumental support (TPxIS), with equal cross-lagged effects over three measurement points. Numbers in squares and circles represent the measurement point. Dotted lines represent correlations and solid lines represent lagged paths. Grey lines represent insignificant paths above *p* > .05, black lines represent significant paths under .05. * *p* < .05, ** *p* < .010, *** *p* < .001. Model fit: □² (df) = 33.53 (26), □²/df = 1.29, RMSEA = .017, SRMR = .020, and CFI = .998

Next, we tested whether equality constraints on the cross-lagged effects were admissible. The crossed-lagged effects were not assumed to vary across the measurement points because the time intervals between all measurement points were of comparable length (ca. four months). Equality constraints would result in a more parsimonious and therefore preferable model (Hamaker et al. 2015). Model comparisons demonstrated that the chi-square differences were not significant (*p* ≥ 0.05), therefore we continued with the more parsimonious RI-CLPMs with equality constraints on the cross-lagged effects. Robust model fit indices demonstrated very good model fit (see Appendix, Figure 3 and 4).

### Intraindividual changes of time pressure, emotional exhaustion, and social support

The within-person level reflected an intraindividual process that captured fluctuations over time of teachers’ perceived time pressure, emotional exhaustion, and received social support from the school principal. Predictors at T1 represented teachers’ deviations from their own expected score instead from the sample mean (Hamaker et al. 2015), which are used to predict deviations from their expected score in time pressure and emotional exhaustion at T2. In turn, T2 deviations were used to predict T3 deviations.

Figure 3 and 4 (see Appendix) show that at the within-person level all cross-lagged effects and most autoregressive effects did not reach statistical significance. In other words, intraindividual changes of teachers’ perceived time pressure and emotional exhaustion could not be reciprocally predicted while controlling for previous levels (i.e., autoregressive effects) and for stable, time-invariant between-person differences across the school year. Therefore, hypotheses 1 and 2 have to be rejected. Social support from the school principal also did not predict intraindividual changes in perceived time pressure and emotional exhaustion, neither as direct effect nor in interaction with perceived time pressure or emotional exhaustion. Concluding, hypothesis 3 has to be rejected. However, it is noteworthy that the receipt of social support from the school principal at T1 predicted an increase in the receipt of social support from the school principal at T2 (*β* = 0.21, *p* = 0.032). This also applies to the effect from T2 on T3 (*β* = 0.16, *p* = 0.045), (see Appendix, Figure 3and 4).

Cross-sectional correlations within one measurement point partly reached statistical significance. Correlations at the first measurement point (T1) indicate the extent to which teachers’ deviations from their own expected score in two variables are related. Correlations at T2 and T3 are characterized as residual correlations and indicate to what extent two variables simultaneously change based on other unobserved variables. In both two models, perceived time pressure and emotional exhaustion at T1 and at T3 were moderately related (*β* = 0.32 at T1 and *β* = 0.34 at T3, *p* < 0.001). The T2 correlation between perceived time pressure and emotional exhaustion did not reach statistical significance (*p* ≥ 0.05).

### Relationships between time pressure, emotional exhaustion, and social support at the between-person level

The random intercepts at the between-person level reflected stable time-invariant differences between teachers across one school year. Standardized beta coefficients in both models demonstrated significant relationships between perceived time pressure, emotional exhaustion, and social support (see Appendix, Figs. 3 and 4). The between-person differences in perceived time pressure and emotional exhaustion reflected a strong relationship (*β* = 0.66, *p* < 0.001). Thus, teachers’ perceived time pressure across one school year coincided to a great extent with the emotional exhaustion they experience across the same school year. We expected this relationship to be dependent on the amount of social support a teacher receives from the school principal. The more social support a teacher received from the school principal, the weaker the relationship between perceived time pressure and emotional exhaustion should be. However, this hypothesized buffering effect of social support could not be demonstrated in both models. Nevertheless, Figs. 3 and 4 (see Appendix) show that the receipt of social support from the school principal was directly related to perceived time pressure and emotional exhaustion (*β* = − 0.19, *p* < 0.001).

## Discussion

As many teachers experience high work-related stress due to time pressure, their health is potentially at risk. Therefore, the present research aimed to test the relationship between teachers’ perceived time pressure and emotional exhaustion as well as buffering effects of received social support from the school principal. Results could neither confirm the hypothesis of reciprocity between teachers’ perceived time pressure and emotional exhaustion nor the buffering effects of received social support from the school principal. However, there are important findings in line with previous research. Although changes within teachers over time could not be determined, present results indicated that differences between teachers in their experience of time pressure and emotional exhaustion are strongly related. Furthermore, also differences between teachers in their receipt of social support from the school principal were related to lower teachers’ perceived time pressure and emotional exhaustion. This supports prior findings among teachers which argued that time pressure represents an important job demand, whereas social support from the school principal represents an important job resource in the teaching profession.

### The relation between time pressure and emotional exhaustion

The present results revealed a strong positive relationship between perceived time pressure and emotional exhaustion which underlines the potential risk of time pressure for teachers’ health (Skaalvik and Skaalvik 2017a; van Droogenbroeck et al. 2014). The positive relationship between perceived time pressure and emotional exhaustion was indicated by the stable, time-invariant components at the between-person level. In other words, the higher teachers are in their perception of time pressure compared to teachers lower in perceived time pressure, the higher their experience of emotional exhaustion across one school year. These stable time-invariant components at the between-person level are sometimes referred to as “trait-like” components (Hamaker et al. 2015). Although perceived time pressure and emotional exhaustion can be in part traced back to certain personality traits (Kokkinos 2007; Sonnentag et al. 2014), we do not consider it as plausible that teachers’ personality traits completely account for their perception of time pressure and experience of emotional exhaustion. Rather, it suggests that in contrast to temporary changes of time pressure and emotional exhaustion, there are enduring, “chronic” aspects of teachers’ daily occupational reality that affect the experience of time pressure and emotional exhaustion.

Further, present results did not show reciprocal effects between perceived time pressure and emotional exhaustion. One reason for this might be that other unobserved variables such as student or classroom characteristics prevent reciprocal effects between perceived time pressure and emotional exhaustion. In support of this suggestion present results showed no relationship over time between intraindividual changes in perceived time pressure and emotional exhaustion, while cross-sectionally they were related. Another reason might be the length of the time intervals between the three measurement points. Possibly they were too short or too long. As theories on temporal processes are basically lacking, research on optimal time lags is inconclusive (Scholz 2019). In the present research the time lags between the measurement points were approximately four months and lagged effects turned out insignificant. In other words, although the intra-class correlations (ICC) indicated a reasonable amount of variance at the within-person level (albeit less than at the between-person level), the model variables were rather stable across the three measurement points. This might have added to the difficulty in finding changes at the within-person level.

In addition, although the present results show that teachers’ perceived time pressure is positively related to emotional exhaustion and negatively to the receipt of social support from the school principal, this does not need to be the complete picture. The challenge-hindrance framework (Lepine et al. 2005) distinguishes between hindrance demands and challenge demands. Hindrance demands have a negative effect on employees’ motivation, satisfaction, and performance whereas challenge demands have a positive impact on employees. Time pressure may also have favourable effects on other unexamined teacher outcome variables. In line with this, previous research supported the dual nature of time pressure (Widmer et al. 2012) by representing a hindrance demand resulting in negative outcomes such as lower well-being and more burnout symptoms, and a challenge demand resulting in positive outcomes such as higher job satisfaction and engagement (Skaalvik and Skaalvik 2017b, 2018).

### The role of social support

Present research showed that received social support from the school principal had a direct negative relationship with teachers’ perceived time pressure and emotional exhaustion—buffering effects could not be found. Although this is in contrast to our hypothesis on the role of social support, it is in line with previous research that showed that social support from the school principal is negatively associated with teachers’ workload, time pressure, and burnout (Dick and Wagner 2001; Skaalvik and Skaalvik 2009). Moreover, harmful consequences of the receipt of social support as previous studies showed (Beehr et al. 2010; Deelstra et al. 2003; Scholz et al. 2012) did not appear in the present results. The absence of buffering effects of the receipt of social support from the school principal corresponds to review studies that show that the empirical evidence for the buffer hypothesis is rather weak and inconsistent (de Lange et al. 2003; Häusser et al. 2010; Mathieu et al. 2019). In our study this might be due to several reasons.

Besides the main findings of the present study an additional finding regarding social support is worth mentioning. The significant autoregressive effects of the receipt of social support from the school principal demonstrated that the more social support a teacher receives from the school principal, the more social support a teacher also receives four months later from the school principal. This is in line with the JD-R model and prior empirical research: Based on the conservation of resources theory (COR theory; Hobfoll 2001) the JD-R model proposes that gaining job resources results in even more job resources due to a reciprocal relationship with work engagement. Job resources are assumed to lead to more work engagement, which vice versa should lead to more job resources (Bakker and Demerouti 2017). This stems from COR theory assumptions that individuals invest resources to protect against loss of resources and to gain resources. Individuals that gain resources are in a better position to invest resources which results in an accumulation described as a gain cycle (Hobfoll 2001). Studies among teachers provided empirical evidence that indicated a gain cycle of job resources (Bakker and Bal 2010; Dicke et al. 2018; Simbula et al. 2011). Thus, the present study adds to these findings and might hint to a gain cycle in the receipt of social support from the school principal.

### Limitations and future research

This study had several limitations. First, although the study had a longitudinal design and the use of a RI-CLPM enabled to detect within-person changes, we cannot draw conclusions about causality. It might be that a third variable explains the relationships found (Mackinnon and Pirlott 2015). Experimental designs in which job characteristics can be manipulated might enhance the possibility of determining causality. Such designs would also assist in further research on the potential reciprocity of the relationship between job demands and health complaints.

Second, the study sample showed moderate levels of perceived time pressure and low levels of emotional exhaustion with little fluctuations during the school year. One likely explanation for this is that teachers with high stress levels did not take part in the study—maybe because they were already too exhausted to participate in this longitudinal study. For future research it is important to discuss how to involve even highly stressed teachers to examine the relationships between job demands and resources.

Third, due to the use of a RI-CLPM, which requires a minimum of three measurement points, we were not able to examine effects from the first measurement point directly on the third measurement point. This might yield different findings, because effects could differ depending on the length of the time interval (Dormann and Griffin 2015). By applying more than three measurement points, this would be possible and simultaneously separate within- and between-person effects.

Fourth, to reduce model complexity we used scale scores of the model variables as observed variables, rather than using the observed indicators. This implies that we did not control for measurement error, which may have affected the results. Because we used only self-reports, it could be argued that the measurement error reflects common method variance (CMV; Podsakoff et al. 2003), although it is contested that CMV poses a major issue (Spector 2006). However, we deliberately chose to use self-reports because we were interested in the personal experience and perception of time pressure, emotional exhaustion, and receipt of social support. It may be difficult for others to report on this and the use of more ‘objective’ indicators also has disadvantages, such as observers’ bias, halo and stereotype effects (Kerlinger and Lee 2000).

Besides these limitations, two methodological contributions follow from strengths of the present research. In line with studies that recommend separating within-person and between-person effects (Curran and Bauer 2011; Kievit et al. 2013), the present study underlines the importance of assessing both levels. By applying a longitudinal multilevel approach with a RI-CLPM we were able to distinguish between interindividual differences and intraindividual changes and reveal different results. Moreover, comparisons with the conventional CLPM indicated that separating between- and within-person effects yields a far better model fit. Therefore, we could draw conclusions at the appropriate analytical level and attain more differentiating results of teachers’ perceived time pressure and emotional exhaustion. Taking these analytical levels and differential results in account substantially adds value to longitudinally designed research. Furthermore, a longitudinal design with three measurement points over the course of one school year gives a detailed insight in the occupational reality of teachers. The work of teachers in Switzerland takes place within a scheduled school year. This results in a relatively stable pattern of periods with higher and lower stress. Therefore, the time frame of one school year is important to examine teachers’ experiences of job resources, job demands, and their well-being. Besides this, the use of a minimum of three measurement points is recommended because a design with two measurement points is regarded as insufficient to detect effects over time (Ployhart and Vandenberg 2010).

### Conclusion

The present research points to the importance of time pressure for teachers’ health and the supportive role that school principals can have in promoting teachers’ health. Overall, the present study provides two main insights for the teaching profession: First, time pressure is an important job demand and strongly related to teachers’ emotional exhaustion. Second, social support from school principals unfolds its function as a resource directly by relating to lower levels of teachers’ perceived time pressure and emotional exhaustion. These results urge schools to consider ways to either limit the time pressure experienced by teachers through organizational measures or organize interventions to perceive time pressure as an aspect that can contribute to feelings of efficacy and competency. Moreover, school principals play an important role in dealing with time pressure and emotional exhaustion by providing social support. This may be by listening to the problems of teachers, encouraging them, showing respect, or helping to complete work or to see opportunities in times of trouble.

## Appendix 1

See Fig. ^3^

## Appendix 2

See Fig. ^4^
